# Understanding Cows’ Emotions on Farm: Are Eye White and Ear Posture Reliable Indicators?

**DOI:** 10.3390/ani9080477

**Published:** 2019-07-24

**Authors:** Monica Battini, Anna Agostini, Silvana Mattiello

**Affiliations:** Dipartimento di Medicina Veterinaria, Università degli Studi di Milano, 20133 Milan, Italy

**Keywords:** positive indicators, emotions, valence, arousal, dairy cows, eye white, ear posture

## Abstract

**Simple Summary:**

It is globally recognized that emotions are important elements of farm animals’ life. However, scientific understanding regarding how to measure and interpret positive emotional states is currently lacking. This study investigated whether eye white and ear posture can reliably help in the interpretation of mood and level of excitement in dairy cows. We found that eye white and ear posture are strongly correlated, and that can be used as complementary measures to interpret emotions. Daily access to pasture has beneficial effects on cows’ emotions. Animals are more relaxed than in any other context, with most of the animals exhibiting half-closed eyes and ears hung down or backwards. The cows were found to be particularly excited during the execution of a human-animal relationship test, showing eye white clearly visible and ears directed forwards, towards the assessor. Housing has an important effect on cows’ emotions: the lower the competition for resources (i.e., in case of more feeding places or cubicles than the number of animals), the lower the level of excitement. This research is a further step towards the use of indicators able to measure emotions in dairy cows and can contribute to enhance animals’ quality of life on farm.

**Abstract:**

Understanding the emotions of dairy cows is primarily important in enhancing the level of welfare and provide a better life on farm. This study explored whether eye white and ear posture can reliably contribute to interpret valence and arousal of emotions in dairy cows. The research was conducted in five Italian dairy farms. Four hundred and thirty-six photographs of cows’ heads were scored (four-level), according to the eye white and ear posture during feeding, resting, pasture, and an avoidance distance test at the feeding rack (ADF test). Eye white and ear posture were significantly correlated and influenced by the context (*P* = 0.001). Pasture was the most relaxing context for cows (67.8% of half-closed eyes; 77.3% ears hung down or backwards). The excitement during ADF test was high, with 44.8% of eye white being clearly visible and ears directed forwards to the approaching assessor (95.5%). Housing and management mostly influenced emotions during feeding and resting (*P* = 0.002 and *P* = 0.001, respectively): where competition for feeding places and cubicles was low, the cows showed the highest percentages of half-closed eyes and ears backwards or hung down. This research supports the use of eye white and ear posture as reliable indicators of emotions in dairy cows.

## 1. Introduction

The use of animal-based indicators to assess the welfare of farm animals is broadly accepted, as they are more indicative than resource-based measures of the actual animal experience [1]. So far, the protocols used to assess the welfare of dairy cows mostly include animal-based indicators that evaluate physical conditions (e.g., body condition score, presence of injuries, lameness) or behaviors (e.g., agonistic interactions, social behavior). Only one indicator, namely the Qualitative Behavior Assessment (QBA), is commonly adopted to evaluate the “Positive emotional state”. QBA relies on the ability of observers to judge and integrate perceived details of animals’ body language and posture into descriptors of low/high arousal and positive/negative valence. Studies that were conducted in different species and contexts (e.g., [2,3,4]) showed that this method is valid and reliable. However, the need for extensive training of the observers and the difficulties in validating QBA in on-farm conditions may impair its use [5,6].

It is now globally recognized by researchers that emotions are part of the complex life of dairy cows [7], and understanding how animals communicate their emotional state or cope with the environment is important in ensuring a better quality of life and high levels of welfare on farm [8]. Consequently, the most recent trends in animal welfare research are now focusing on the investigation of valid and feasible indicators that are able to measure valence and arousal of emotions in dairy cows [5]. According to dimensional theories [9], emotions can be described as moving in a *continuum* along two axes: valence, which expresses the mood (positive or negative), and arousal, which defines the level of excitement (low or high). Obviously, valence and arousal are strictly linked, and possible interactions need to be considered [10]. Negative emotions often coincide with high arousal (e.g., separation from a group [10]), but high arousal can also be found during positive situations (e.g., receiving highly palatable food [11]), and positive emotions are often characterized by low arousal (e.g., grooming [7]).

To date, many studies have focused on eye white and ear posture as potential promising indicators for interpreting emotions in dairy cows. The majority of research that has been performed to explore the reliability of eye white as an indicator of emotional state was performed on cows (e.g., [7,11,12,13,14,15,16]), but few studies have also been conducted on sheep [10,17,18,19]. Research conducted so far confirms the ability of this indicator to capture the variations of arousal of animals that were subjected to different stimuli: eye white percentage or eye aperture significantly decrease when cows and sheep experience a low arousal event, usually with a positive valence, such as gentle stroking [7,10]; conversely, they increase during high arousal situations with either negative (suddenly opened umbrella [13], denied access to visible food [14], calf-cow separation [15], separation from the group [10], being fed with inedible woodchip [11,20]) or positive valence (highly desirable concentrate feed [11]). Only one study does not report differences in the percentage of visible eye white depending on the context (feeding *versus* claw trimming in dairy cows) [21]. However, the authors found a possible confounding breed effect that may have masked the potential treatment effect: this might be due to differences in eye coloration patterns, with Red Holstein cows having more contrast between eye white and iris and Brown Swiss having less contrast due to their darker eye white [21].

Animals use a complex set of body postures and facial expressions to communicate their emotions. In particular, ear postures are largely adopted by a wide range of farm animals in social communication and to express internal states [22]. Ear postures can reveal different emotions, depending on the species; hence, specific studies are needed to gather species-specific information from this indicator [23]. Ruminants have highly developed muscles around their ears, which enables them to independently move ears in many different ways [17]. Therefore, the study of ear posture as an indicator of emotions seems particularly promising. Studies on ear posture have been conducted in dairy cows, as well as in other ruminants (sheep [17,24,25,26], goats [27]). Backwards or hanging ears in dairy cows and sheep are associated with the positive emotional states of low arousal, as might be induced by stroking or grooming [23,28,29]; consistently, goats spend more time with ears in a forward position when they observe pictures of other goats’ faces that were taken in negative situations [27].

This work aimed to investigate whether the visible eye white and ear posture can reliably contribute to interpret the valence and the arousal of emotions in dairy cows. First, we investigated the relationship between eye white and ear posture. Secondly, each indicator was evaluated in different contexts (feeding, resting, pasture, and avoidance distance test at the feeding rack) that are supposed to elicit different emotions. Finally, within the same context, we investigated the effect of different housing and management conditions on the emotional state of dairy cows.

## 2. Materials and Methods

The study was conducted from March to June 2018 in five dairy farms that were located in Northern Italy. Table 1 presents the main farm characteristics.

More than 500 photographs of cow’s heads were taken by the same assessor from one random side of each cow in four different contexts: (1) feeding (head in the feeding rack), (2) resting (cows lying down, either sleeping or ruminating), (3) pasture (cows engaged in different activities while at pasture, such as grazing or lying; this context could be observed only in farm 5, where pasture was available), and (4) an avoidance distance test at the feeding rack (ADF test). The test was performed by a person that was unknown to the animals, who stood still at 200 cm distance in front of each cow at the feeding rack (not restrained) and slowly approached the animal with the arm lifted (45°) and the hand palm directed downwards after having established a reciprocal visual contact, until the first avoidance reaction of cows [30]. ADF test could be performed only in farm 1, thanks to the simultaneous presence of two assessors: one performing the test, the other one taking photographs. Resting was never observed in Farm 2, due to the limited observation time.

**Figure 1:**
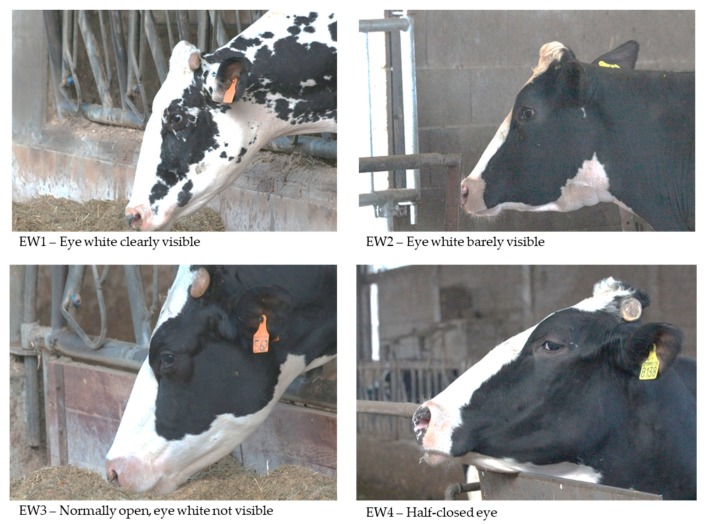
Eye white classification.

**Figure 2:**
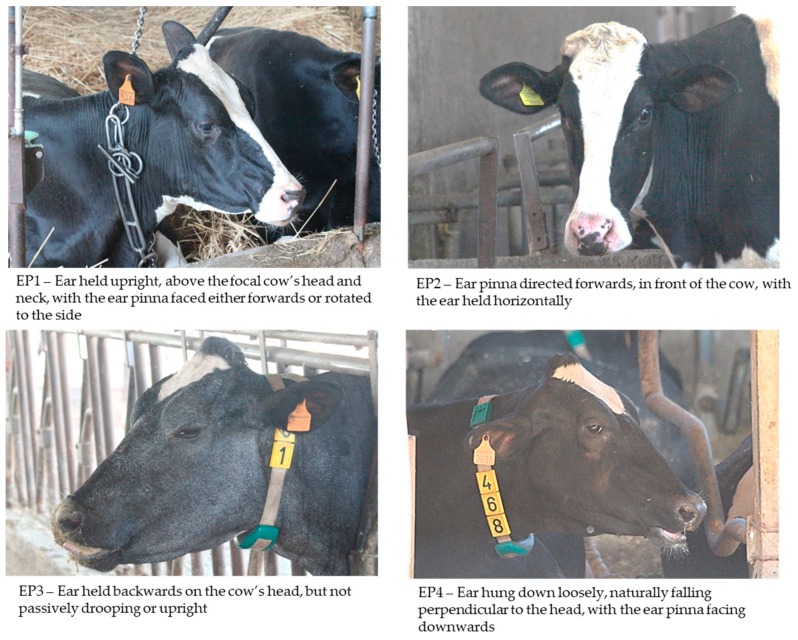
Ear posture classification (previously defined by Proctor and Carder, 2014 [23]).

The photographs were taken from a distance while using a Canon 650d, mounting a Canon EF 70–300 mm f/4-5.6 IS USM II telephoto lens in order to minimize the assessor’s effect on the animals. The photographs were scored from 1 to 4 according to eye white and ear posture, as described in Figure 1; Figure 2. For eye white, we proposed this four-level classification based on eye aperture and if the white was visible or not, instead of the computerized measurement that was proposed by Sandem et al., 2002 [7], in order to improve the feasibility of this indicator. The classification adopted for ear posture is the same as described by [23]. For both of indicators, the lower scores correspond to the highest level of arousal/excitement, and vice versa.

A non-parametric Spearman rank correlation test was used to measure the degree of association between the eye white and ear posture scores. A Chi Square test was used for testing the differences in the proportion of eye white and ear posture classes among contexts, and among farms within each context (only feeding and resting contexts were considered for this last analysis, as the pasture and ADF test could only be recorded in farms 5 and 1, respectively).

## 3. Results

Some of the photographs of cows’ heads had to be discarded due to the incomplete visibility of eyes or ears. A total of 436 photographs were retained for eye white classification (feeding = 129; resting = 181; pasture = 59; ADF test = 67) and 489 photographs were used for ear posture classification (feeding = 137; resting = 219; pasture = 66; ADF = 67). Only 429 photographs allowed for the analysis of correlation between eye white and ear posture, as both the eye and ear were clearly visible.

**Figure 3:**
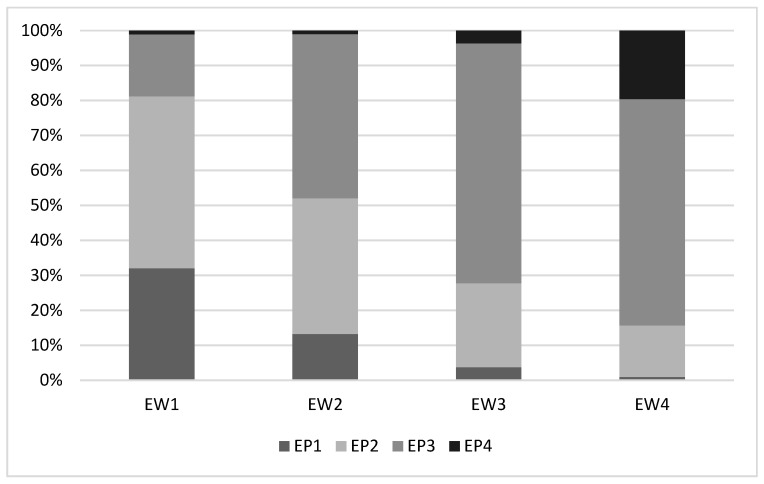
Association between eye white and ear posture: the highest percentage of eye white clearly visible corresponds to the highest percentage of ear in upright position, and to the lowest percentage of ear hung down loosely.

Eye white and ear posture were clearly associated: a high portion of visible white corresponded to ears held upright or directed forwards, whereas half-closed eyes corresponded to ears that were held backwards or loosely hung down (Figure 3); the correlation between eye white and ear posture scores was statistically significant (Spearman rank correlation test: ρ = 0.570; *P* = 0.001).

**Figure 4:**
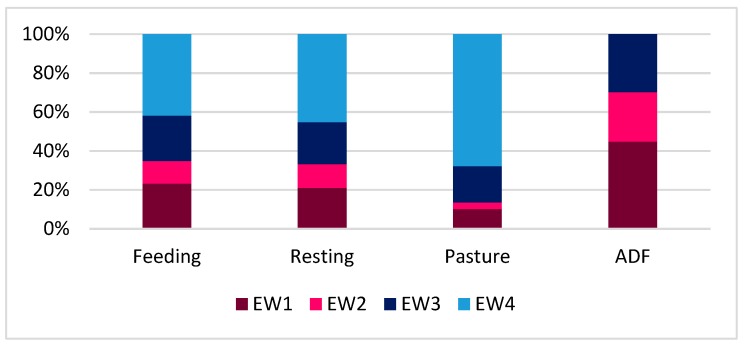
Percentage distribution of the four eye white classes in each context (feeding, resting, pasture, ADF–avoidance distance test at the feeding rack).

**Figure 5:**
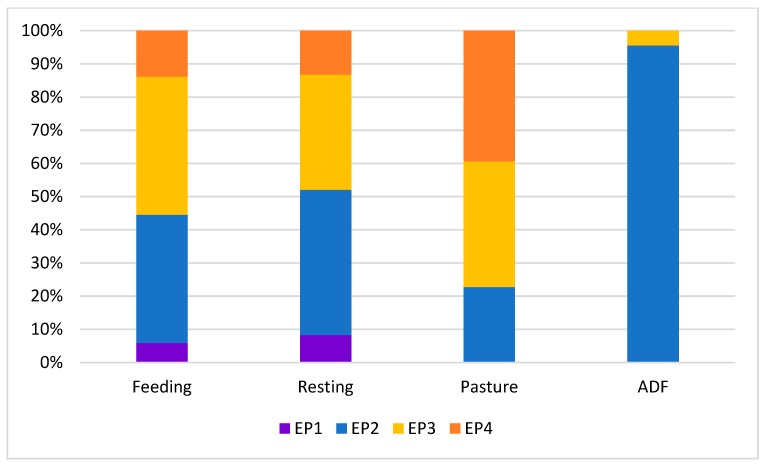
Percentage distribution of the four ear posture classes in each context (feeding, resting, pasture, ADF–avoidance distance test at the feeding rack).

The visible eye white and ear posture were both significantly influenced by context (*P* = 0.001; Figure 4 and Figure 5).

In particular, the highest percentage of half-closed eye (EW4) was associated to the low arousal and positive experience of pasture (67.8%), followed by resting (45.3%) and feeding (41.9%). Half-closed eye was never observed during the ADF test. During the ADF test, the highest percentage of eye white clearly visible (EW1 = 44.8%) was recorded; the highest percentage of cows with normally open eye (EW3) was recorded during the ADF test (29.8%).

During pasture, most of the cows loosely held the ears down (EP4 = 39.4%) or backwards (EP3 = 37.9%); most of the cows held the ears backwards (EP3 = 41.6%) or with the ear pinna directed forwards (EP2 = 38.7%) during feeding. During ADF, nearly the totality of cows directed the ear pinna forwards (EP2 = 95.5%). The cows only showed ear upright (EP1) during feeding (5.8%) and resting (8.2%).

The more common contexts, i.e., feeding and resting, were further analyzed in order to highlight the differences among farms. For eye white, statistical differences among the farms were observed for both feeding (*P* = 0.002) and resting (*P* = 0.001).

**Figure 6:**
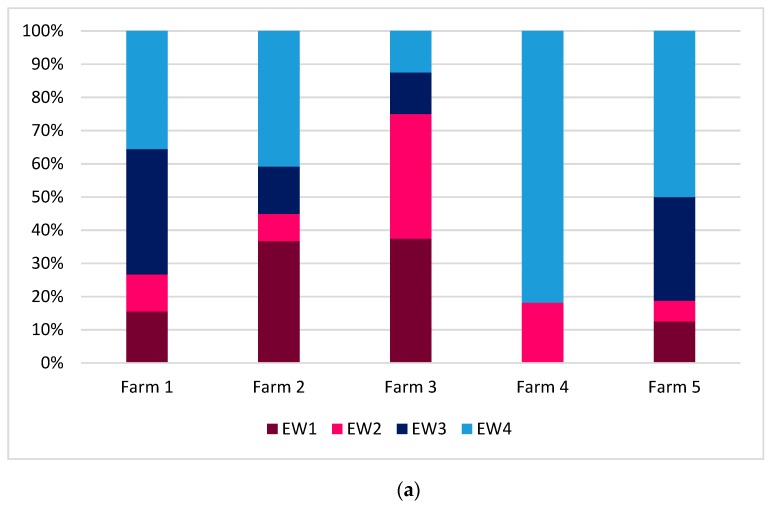

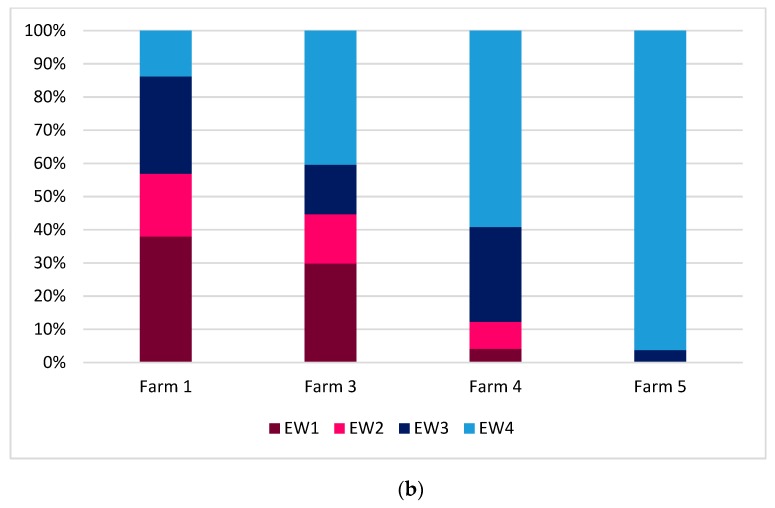
Results of the eye white in the farms included in the study. (**a**) during feeding; and, (**b**) during resting.

Most of the cows in farm 4 showed half-closed eye during feeding (EW4 = 81.8%); the cows in farm 5 showed eye from half-closed to normally open (EW4 = 50%; EW3 = 31.2%). The cows in farm 3 showed eye white ranging from barely to clearly visible (37.5% for both EW2 and EW1) (Figure 6a).

During resting, nearly the totality of cows showed half-closed eye during resting in farm 5 (EW4 = 96.3%), whereas the highest percentages of eye white clearly visible (EW1) was recorded in Farm 1 (37.9%) and 3 (29.8%) (Figure 6b).

**Figure 7:**
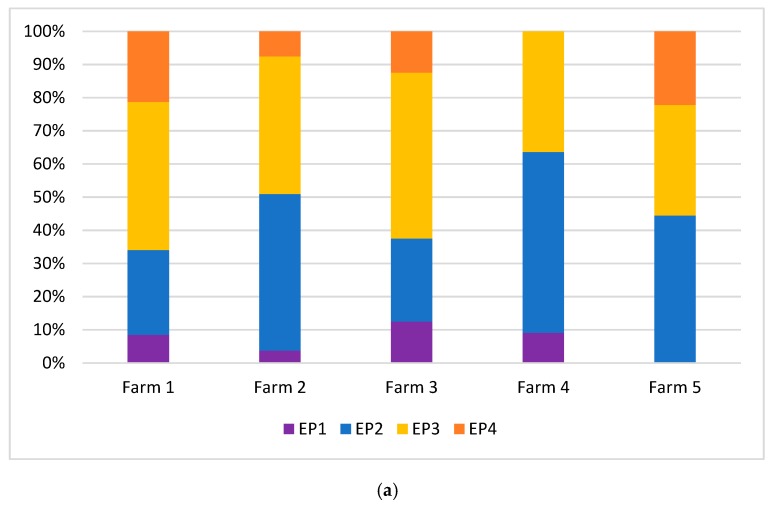

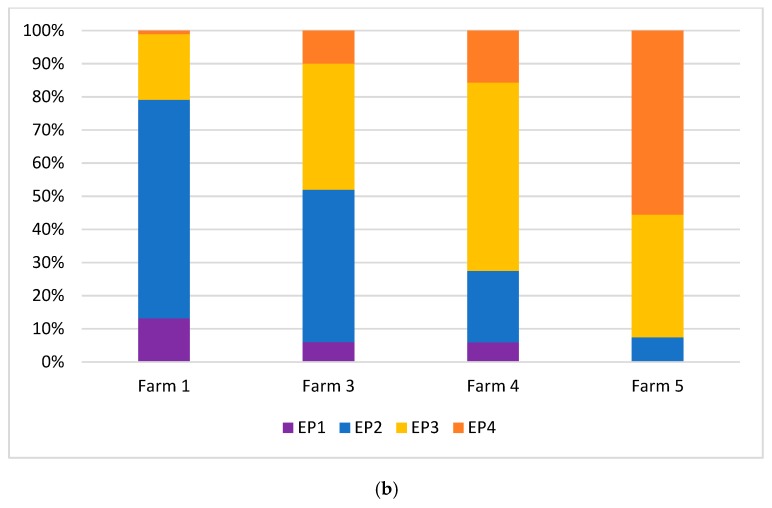
Results of the ear posture in the farms included in the study. (**a**) during feeding; (**b**) during resting.

As to ear posture, no statistical difference was found among farms during feeding, as in all the farms most of the cows generally held the ear backwards (EP3) and forwards (EP2) (Figure 7a). However, it is worth noticing that the highest percentage of cows with ears held backwards was recorded in farm 3 (EP3 = 50%) and the highest percentage of cows with ears held forwards was found in farm 4 (EP2 = 54.5%). The cows rarely held the ear in upright position during feeding, except in farm 3 (12.5%; Figure 7a).

Ear posture statistically differed among farms during resting (*P* = 0.001). Farm 5 showed the highest percentage of cows with ears loosely hung down (EP4 = 55.5%), and no cow held the ears upright (EP1 = 0%); farm 4 had the highest percentage of cows with ears held backwards (EP3 = 56.9%); in farm 1, the highest percentage of cows held the ears forwards (EP2 = 65.9%) and upright (EP1 = 13.2%) (Figure 7b).

## 4. Discussion

Our results show that taking photographs from a distance directly on the farm can be an easy way to collect reliable information regarding eye white and ear posture. This method is quicker and more feasible than video recording. On the other hand, this method does not allow for collecting changes in ear posture that resulted as relevant in some studies in sheep and lambs (e.g., [10,17,24,25]), but whose interpretation provided contradictory results in dairy cows (e.g., [22,23]). We also excluded the collection of asymmetric ear posture, which was frequently found during social isolation in sheep [17]. The decision to take photographs from one random side was due to our aim to find possible associations between eye white and ear posture taken together on the same animal in different contexts. To the best of our knowledge, this is the first attempt to find this association. Regarding on-farm feasibility of eye white, it also has to be remarked that the use of the four-level classification proposed in the present study, with the aid of a telephoto lens (70–300 mm), made this indicator more feasible than using the computerized approach that was proposed by Sandem et al., 2002 [7]. Therefore, this simplified scale proved to be both valid and practical for on-farm assessment.

Our results confirm that cows simultaneously and consistently use different parts of their head to communicate their emotions. Half-closed eyes are mostly associated to ears hanging down, whereas when the eye white is visible, the ears are often held forwards or upright.

Our study was conducted in commercial farms, hence the situations to which cows were exposed were part of their daily life, with the only exception of the ADF. Pasture was the context in which cows showed the lowest arousal (eyes were half-closed in 67.8% of cows) and probably with positive valence (ears hanging down or backwards in 77.3% of cows). Access to pasture has many beneficial effects on the welfare of dairy cows [31], such as a reduction of mortality rate, mastitis, reproductive problems and lameness, and an increased possibility to move, to rest in comfort, to eat preferable food, and to express species-specific behaviors. For these reasons, cows highly prefer outdoor to indoor housing (for review [32]). It is also supposed that sunlight exposure is rewarding and that cows can derive welfare benefits when they are outdoors [32]. However, we also found 37.9% of cows with ear held forwards. This result suggests that pasture may be both a low arousal situation and a stimulating context where cows actively direct their attention to the surrounding.

It is partly surprising that the level of arousal during pasture is even lower than during the sole resting, although the positive effect of the access to pasture is well recognized (half-closed eyes 67.8% versus 45.3%); in addition, we found 8.2% of cows with ear upright during resting, which confirmed that the level of excitement was high in this context for some animals. We can also hypothesize a negative emotional valence expressed by cows that show this ear posture. Resting can be affected by several factors, such as the cubicle: cow ratio, cubicle design (e.g., length, width), housing system (tethered vs loose housing), etc. In farms where the competition for the resting area was high (as in farm 1, cubicle: cow ratio = 0.89), most of the cows had the eye white clearly visible and the ears held forwards and upright (e.g., the highest percentage of ear in upright position was recorded in farm 1: EP1 = 13.2%). We can suppose that the cows were alerted (high arousal) and nervous (negative valence) during social interactions in highly competitive contexts. In farm 3, where cows were in tie stalls, 44.7% of cows showed eye white from barely to clearly visible, 46% of ear held forwards, and 6% of ears in upright position. This husbandry system is likely to be less comfortable than loose housing, as confirmed by several authors [33,34,35,36], which was mainly due to the increased risk for lesions and lameness, and to the restriction of voluntary movements and social behavior. We might hypothesize that being tethered in a tie stall is not relaxing for cows: in fact, [36] observed that lying down position was frequently incorrect in tie-stalls, where almost 35% of cows laid partly or completely outside the stall, or showed evident signs of compression or discomfort of the hind part of the body. Hence, we can suppose that the underlying emotional valence expressed by cows was negative, which was possibly due to discomfort rather than to competition. In farm 5, where the cubicle: cow ratio was positive (1.12), and the cows also had access to additional outdoor space, we found the more relaxed cows (low arousal, positive valence), as shown by the highest percentage of half-closed eyes (96.3%) and hanging ears (55.5%). We hypothesize that, when cows live in environments where the competition for resources is limited, the arousal is low and is probably associated to a positive valence. An additional hypothesis is that pasture may have a long-lasting effect on cows’ emotional state, which goes beyond the bare time spent outdoors, and leads to a general more positive state even when animals are indoors.

In our research, during feeding cows were particularly excited (34.9% of cows with eyes from barely to clearly visible; 5.8% with ears in upright position), which is in agreement with other studies showing that feeding produces greater arousal than other activities (e.g., rumination [10]). As already discussed for resting, feeding can be a competitive context and emotions can be influenced by several factors, such as feed quality and space at the rack. Cows seem to be more at ease (positive valence) in farms where the feeding space: cow ratio is at least 1, as in farm 4, or 1.72 as in farm 5, that is where the competition is low. This is supported by the highest percentage of eyes from half-closed to normally open (81.8% in farm 4; 81.2% in farm 5) and, in farm 5, also by the high percentage of cows with ears that were held backwards or hung down loosely, which indicate a positive state that was also probably due to the type of feed (ventilated hay and fresh grass, instead of total mixed ration). This result is in line with a study conducted in sheep fed fresh hay that revealed a high proportion of animals with ears in the backward position [17]. Even for feeding, as for resting, we cannot exclude that this positive emotional state is also partly due to the long-lasting positive effect of the access to pasture. In farm 3 (tie stalls), the feeding place: cow ratio was obviously 1. However, 75% of the cows showed eye white from barely to clearly visible, and the highest percentage of cows with ear upright (12.5%) was recorded. We can suppose that the limited possibility to move during feeding and the frustration due to the reduced possibility to solve social conflicts with neighboring cows may represent a stressful situation (negative valence) even when cows have sufficient space to access the feed rack. Differently, in farm 4, where the feeding place: cow ratio is also 1, the ears are frequently held forwards (54.5%). Studies on goats [37] revealed that ears forwards can be a sign of vigilance: hence, the arousal is high, but valence may be either positive or negative, and expresses attention to the surrounding rather than stress.

ADF elicited the strongest reaction in cows: eye white clearly visible was mostly recorded during the execution of this test (44.8%). As a high percentage of eye white may be a valid indicator of fear in dairy cows [13], we may suppose that, during the execution of the test, cows are experiencing emotions with a negative valence (i.e., fear) and probably a high arousal. Unsurprisingly, during ADF test, most of the cows directed the ear pinna forwards towards the assessor (95.5%), which confirmed that an approaching person could cause a vigilance reaction in cows. Further studies would be interesting in order to match eye white and ear posture scores to the results of the ADF test for each cow, to gather more information regarding the perception of danger and the quality of the human-animal relationship.

Some of the studies reported that eye white can better express the arousal of emotions [23], whereas the valence can also affect ear posture [22]. Even if both indicators can help interpreting emotions in dairy cows in the same direction, the two measures seem complementary, as eye aperture and ear movements can have different meanings for a cow.

## 5. Conclusions

This research confirms that eye white and ear posture can be promising indicators to be included in the assessment of emotions of dairy cows on farm. However, even though cows show a rather consistent use of eyes and ears to communicate their emotions, the correlation between these two indicators is not completely linear, and a combined use of eye white and ear posture would probably give a more complete and reliable picture of the valence and arousal that were experienced by the animals.

The collection of these indicators in contexts that are routinely experienced by cows during their daily life can contribute to adding information regarding the perception of the environment and the valence and the arousal of emotions in different situations, but the reliability of these indicators should be further investigated, and their feasibility may be improved by collecting data by direct observations.

## Figures and Tables

**Table 1:** Main characteristics of dairy farms involved in the study.

Farm ID	Number of Lactating Cows	Breeds	Husbandry System	Cubicle: Cow Ratio	Feeding Place: Cow Ratio	Feeding	Access to Pasture
1	112	Holstein	Loose house, cubicles	0.89	0.69	Total mixed ration	No
2	120	Holstein	Loose house, cubicles	1.08	0.68	Total mixed ration	No
3	49	Holstein	Tie stall	1.00	1.00	Total mixed ration	No
4	52	Holstein	Loose house, cubicles	1.15	1.00	Total mixed ration	No
5	50	80% Pezzata Rossa Italiana, 20% Holstein	Loose house, cubicles	1.12	1.72	Ventilated hay and fresh grass	Yes
